# Traumatic Brain Injury Modifies the Relationship Between Physical Activity and Global and Cognitive Health: Results From the Barcelona Brain Health Initiative

**DOI:** 10.3389/fnbeh.2019.00135

**Published:** 2019-06-19

**Authors:** Timothy P. Morris, Jose-Maria Tormos Muñoz, Gabriele Cattaneo, Javier Solana-Sánchez, David Bartrés-Faz, Alvaro Pascual-Leone

**Affiliations:** ^1^Institut Guttmann, Institut Universitari de Neurorehabilitació Adscrit a la UAB, Badalona, Spain; ^2^Universitat Autònoma de Barcelona, Bellaterra, Spain; ^3^Fundació Institut d’Investigació en Ciències de la Salut Germans Trias i Pujol, Barcelona, Spain; ^4^Berenson-Allen Center for Noninvasive Brain Stimulation, Division of Cognitive Neurology, Department of Neurology, Beth Israel Deaconess Medical Center, Harvard Medical School, Boston, MA, United States; ^5^Departament de Medicina, Facultat de Medicina i Ciències de la Salut, Universitat de Barcelona, Barcelona, Spain; ^6^Institut d’Investigacions Biomediques August Pi i Sunyer (IDIBAPS), Barcelona, Spain

**Keywords:** physical activity, cognition, global health, traumatic brain injury, health-related quality of life

## Abstract

Physical activity has many health benefits for individuals with and without history of brain injury. Here, we evaluated in a large cohort study the impact of physical activity on global and cognitive health as measured by the PROMIS global health and NeuroQoL cognitive function questionnaires. A nested case control study assessed the influence of a history of traumatic brain injury (TBI) on the effects of physical activity since underlying pathophysiology and barriers to physical activity in individuals with TBI may mean the effects of physical activity on perceived health outcomes differ compared to the general population. Those with a history of TBI (*n* = 81) had significantly lower Global health (*β* = −1.66, *p* = 0.010) and NeuroQoL cognitive function (*β* = −2.65, *p* = 0.006) compared to healthy adults (*n* = 405). A similar proportion of individuals in both groups reported being active compared to being insufficiently active ($X_{\left(1\right)}^{2}$ = 0.519 *p* = 0.471). Furthermore, the effect of physical activity on global health (*β* = 0.061, *p* = 0.076) and particularly for NeuroQoL (*β* = 0.159, *p* = 0.002) was greater in those with a history of TBI. Individuals with a history of TBI can adhere to a physically active lifestyle, and if so, that is associated with higher global and cognitive health perceptions. Adhering to a physically active lifestyle is non-trivial, particularly for individuals with TBI, and therefore adapted strategies to increase participation in physical activity is critical for the promotion of public health.

## Introduction

Physical activity is associated with a 20%–30% lower risk in all-cause mortality and incidence of multiple chronic conditions (James et al., 2016). Numerous governing bodies including the World Health Organization, American Heart Association and the American College of Sports Medicine have focused much attention on the beneficial effects of a physically active lifestyle (Warburton et al., 2006; Haskell et al., 2007; WHO, 2019a). The effects span multiple bodily systems from cardiovascular benefits (James et al., 2016) to mental health (Blumenthal et al., 1999; Babyak et al., 2000) and cognitive function (Colcombe and Kramer, 2003; Gomes-Osman et al., 2018; Northey et al., 2018). The effects of physical activity on cognitive function has received particular attention in recent decades across distinct age groups, including adolescents (Donnelly et al., 2016), older adults (Colcombe and Kramer, 2003) and patients with dementia (Gomes-Osman et al., 2018). Physical activity represents a modifiable lifestyle factor capable of improving global and cognitive health across the lifespan. However, engaging in a physically active lifestyle is non-trivial. Globally, one in four adults are classified as insufficiently active (WHO, 2019b), which is estimated to contribute to 9% of all premature deaths worldwide (or 5.3 million; Lee et al., 2012). Physical inactivity is exacerbated in those with a disability, with almost half of US adults with a disability being physically inactive and having a greater likelihood of having a chronic disease (Carroll et al., 2014).

Individuals who have acquired brain injuries can become prone to a sedentary lifestyle which places them at risk of secondary health complications (Shavelle et al., 2001). A number of studies have reported that individuals with a history of traumatic brain injury (TBI) are insufficiently active enough to receive a health benefit (Reavenall and Blake, 2010; Hamilton et al., 2015), according to current guidelines (Larry Durstine et al., 2012). The long-term health consequences of TBI include cognitive, sensorimotor, behavioral and social problems that can negatively affect quality of life (Stocchetti and Zanier, 2016), and in the US alone, an estimated 3.2 million individuals live with residual effects of TBI (Benedictus et al., 2010). Cognitive dysfunction following TBI can last for decades post-injury (Draper and Ponsford, 2008) and physical exercise is a potential therapeutic intervention for those living with residual effects of an injury (Hoffman et al., 2010; Wise et al., 2012; Chin et al., 2015). Whilst the feasibility of dedicated and professional-led, short-to-mid-term (3 months) aerobic exercise programs in community-dwelling individuals with moderate-to-severe TBI has been demonstrated (Devine et al., 2016), the association between a physically active lifestyle and global and cognitive brain health in this population has not been assessed. Distinct barriers to performing physical activity (Rimmer et al., 2004) and underlying pathophysiology (Werner and Engelhard, 2007) may mean that this relationship differs in individuals with a history of TBI.

We performed a nested case-control study to assess the impact of a history of TBI on the associations between physical activity and perceived global and cognitive health. We hypothesized that physical activity would be predictive of higher global and cognitive health in both those with and without a history of TBI.

## Materials and Methods

### Study Design

A cohort of community-dwelling adults, mainly in the Catalonia region of Spain, was established as part of the Barcelona Brain Health Initiative^1^, starting in 2017. Adults aged 40–65 were invited to participate in an online questionnaire-based survey *via* television and local advertisements. The study protocol is described in Cattaneo et al. (2018). All methods described were approved by the education and ethics committee of Institut Guttmann. This manuscript was prepared under the strengthening the reporting of observational studies in epidemiology (STROBE) guidelines.

At the time of analysis (June 2018), a total of 4,624 individuals had enrolled. Of those, 81 individuals (1.8%) answered positively to the question: *Have you ever had a TBI with loss of consciousness?* A control group (*N* = 405, 49% female) was randomly selected from the total cohort using Microsoft Excel’s “rand” function. The total cohort was organized by this random number sequence and then filtered per the age and gender criteria. Whereby five adults free from any neurological or psychological disorders were selected for every participant with a history of TBI at random in blocks of 5 years with an even male to female ratio.

### Outcomes and Covariates

Our main outcome variables were perceived global health and perceived cognitive function. To measure these constructs, the PROMIS global health questionnaire (Cella et al., 2010) and the NeuroQoL cognitive function questionnaire (Gershon et al., 2012) were used. PROMIS global health is a 10-item 5-point Likert scale (1-poor, 5-excellent) that probes respondents physical, mental and social health. Higher scores mean more of the construct is being measured (i.e., higher global health). NeuroQoL cognitive function is 5-point Likert scale (1-very often, 5-never) with 12 items that probes respondents thinking, attention, planning, new task learning and comprehension. Higher scores mean more of the construct is being measured (i.e., better perceived cognitive function).

Our main predictor variable was physical activity levels. We chose to implement the Godin-Shepard leisure time physical activity questionnaire (Godin and Shephard, 1985) to measure this. The GSLTPAQ probes the number of times spent performing moderate (not exhausting) or strenuous (heart beats rapidly) physical activity of at least 15-min during a typical 7-day period (Godin, 2011). The frequency score is multiplied by a corresponding metabolic equivalent for task (MET) value (moderate = *5; strenuous = *9) and summed to obtain an arbitrary leisure score index (LSI). Cut-off points can be created using the LSI, the rationale for which originate from the World Organization (WHO, 2018) and American College of Sports Medicine (Ferguson, 2014) guidelines for weekly physical activity associated with significant health benefits (combination of moderate and strenuous activity 3–5 times per week). An LSI of ≥24 is *active* where as those ≤23 are *insufficiently active*. Consequently, those culminating in a score of ≥24 using questions that pertain to moderate and strenuous physical activity and LSI calculations based on both frequency and energy expenditure will likely meet the physical activity guidelines. The utility and accuracy of these cut-off scores have been validated in healthy adults (Amireault and Godin, 2015). Raw scores above 7 for each question were excluded (*N* = 13) as they were considered to be derived through misinterpretation of the question. Those without responses were set to missing (*N* = 12).

Co-variates included gender and age, which was asked in years and three categories were created; 40–49, 50–59 and 60 and above. BMI was calculated as body weight (in kilograms) divided by height (in meters squared). Self-perceived negative affect in depression, anxiety and stress was also assessed using the 21-item sub-scale version of the Depression Anxiety Stress Scale (Brown et al., 1997). This is a 4-point Likert scale (1-never, 4-always) where higher scores represent higher negative affective state. This was chosen as a potential confounder as perceived negative effect can affect how one reports cognitive and global health (Bartrés-Faz et al., 2018).

### Statistical Analysis

We tested age, gender and negative affective status as potential confounding variables in our analysis. Variables which predicted the outcome measure with a *p* ≤ 0.05 were defined as confounders and added to the final model. The proportion of individuals who reported being insufficiently active compared to active was tested using Pearson’s chi squared test of proportions, as was gender. A Kruskal-Wallis test was used to assess differences in BMI between groups. Generalized linear models with a Gaussian family function and identity link function were used to assess the independent associations between physical activity levels and diagnosis history (history of TBI or not) on perceived cognitive function and perceived global health, controlling for all other significant variables in the model. To assess whether the associations between physical activity and perceived cognitive and global health differed between those with and without a history of TBI, an interaction term between physical activity and diagnosis history was added to the model. Post estimation tests were performed using marginal effects and the estimated slopes were plotted. All statistical analyses were performed in Stata version 15 (StataCorp LLC, College Station, TX, USA).

## Results

Table 1 describes all participant characteristics and questionnaire scores for each group. There was no significant difference between the age of the participants in each group (*t*_(116)_ = −0.3, *p* ≥ 0.740) and the proportion of male and female participants was similar across groups [49% female ($X_{\left(1\right)}^{2}$ = 0.004 *p* = 0.951)]. BMI was not significantly different between each group ($X_{\left(1\right)}^{2}$ = 0.042 *p* = 0.838).

**Table 1:** Participant characteristics and questionnaire scores between groups.

	Healthy adults	History of TBI
Age (years)	51.8 (7.2)	51.7 (7.1)
BMI (kg/m^2^)	24.2 (3.5)	24.4 (4.0)
PROMIS Global Health	34.9 (4.6)	32.2 (6.2)*
PROMIS NeuroQoL	52.3 (6.5)	47.5 (11.0)*
DASS21	13.4 (11.3)	20.4 (17.5)*
GSLTPAQ	19.5 (19.2)	18.8 (18.3)

*Significant at *p* < 0.05. GSLTPAQ, Godin-Shepard leisure time physical activity questionnaire.

### Physical Activity Level

**Figure 1:**
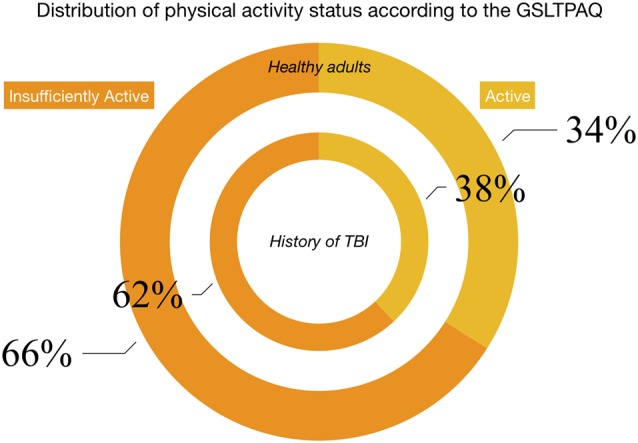
Hollow pie charts show the proportion of individuals who report being active (yellow bars) compared to insufficiently active (orange bars) based on the Godin-Shepard leisure time physical activity questionnaire (GSLTPAQ). The Godin questionnaire classifies people as *active* or *insufficiently active* based on the number of times per week spent performing either moderate or strenuous physical activity of at least 15 min. The inner bars represent the group with a history of traumatic brain injury (TBI) and the outer bars represent those free from any neurological or neuropsychiatric disease. A numerically higher proportion of individuals in both groups report being *insufficiently active* compared to those reporting being *active*.

Using the LSI cut-off scores to classify participants into *active* and *insufficiently active*, a similar proportion of those with and without and history of TBI were classified as *active* ($X_{\left(1\right)}^{2}$ = 0.519 *p* = 0.471), compared to *insufficiently* *active* (Figure 1).

### PROMIS Global Health

**Figure 2:**
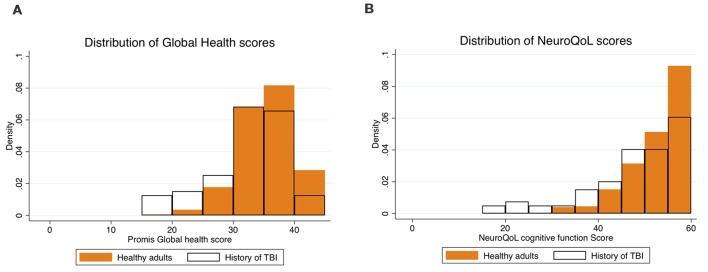
Histogram plots show the distribution of scores on the global health scale **(A)** and the NeuroQoL cognitive function scale **(B)** for both participants in the healthy group (orange filled bars) and the group with a history of TBI (black outlined bars).

**Figure 3:**
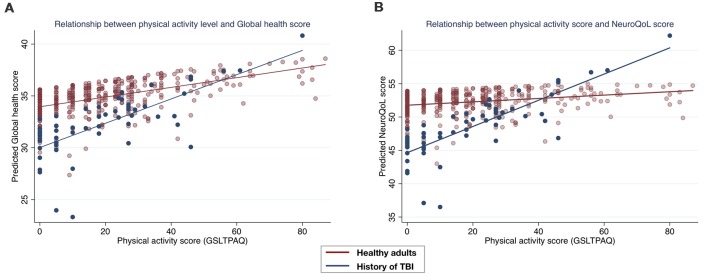
Scatter plots of the relationship between physical activity levels and the predicted Global health and NeuroQoL cognitive function scores as a result of the generalized linear models, by group. **(A)** A trending interaction (*p* = 0.076) was observed between groups whereby the group with a history of TBI had a greater effect of physical activity level on global health (navy line). In **(B)** a significant interaction between groups is observed (*p* = 0.002) whereby the group with a history of TBI shows a significant effect of physical activity on NeuroQoL cognitive function, which is not significant for the group without a history of TBI (red line).

The distribution of Promis global health scores in both groups is shown in Figure 2A. Diagnosis was a significant predictor of global health (*β* = −1.66, SE = 0.644, *p* = 0.010, 95% CI’s = −2.927, −0.403) with those with a history of TBI reporting lower perceived global health (32.2 ± 0.70) than those without (34.9 ± 0.22). Physical activity level significantly predicted Promis global health (*β* = 0.049, SE = 0.011, *p* ≤ 0.001, 95% CI’s = 0.027, 0.071). A non-significant interaction between diagnosis and physical activity level was shown (*β* = 0.061, SE = 0.034, *p* = 0.076, 95% CI’s = −0.006, 0.128). Post estimation tests showed that the relationship between physical activity and global health was marginally greater in the group with a history of TBI (*β* = 0.103, SE = 0.032, *p* = 0.001, 95% CI’s = 0.040, 0.166), than in the healthy adults (*β* = 0.042, SE = 0.011, *p* ≤ 0.001, 95% CI’s = 0.019, 0.065; Figure 3A).

### NeuroQoL Cognitive Function

The distribution of NeuroQoL cognitive function scores in both groups is shown in Figure 2B. Diagnosis was a significant predictor of NeuroQoL cognitive function (*β* = −2.65, SE = 0.974, *p* = 0.006, 95% CI’s = −4.561, −0.743), with those with a history of TBI reporting lower perceived cognitive function health (47.45 ± 1.20) than those without (52.26 ± 3.29). Physical activity level was predictive of NeuroQoL cognitive function (*β* = 0.037, SE = 0.016, *p* ≤ 0.023, 95% CI’s = 0.005, 0.070). A significant interaction between diagnosis and physical activity level was shown (*β* = 0.159, SE = 0.051, *p* = 0.002, 95% CI’s = 0.058, 0.260) whereby post estimation tests showed that the relationship between physical activity and NeuroQoL cognitive function was significant in the group with a history of TBI (*β* = 0.179, SE = 0.049, *p* ≤ 0.001, 95% CI’s = 0.083, 0.274), but not in the healthy adults (*β* = 0.019, SE = 0.017, *p* = 0.260, 95% CI’s = −0.014, 0.054; Figure 3B).

## Discussion

In this study, we aimed to assess the impact of a history of TBI on the relationship between physical activity levels and global and cognitive brain health. Not surprisingly, we found that healthy adults reported higher global and cognitive brain health compared to those with a history of TBI. However, we also found that individuals with a history of TBI were as likely to be physically active as those without a history of TBI and physical activity levels were even stronger predictors of both global and cognitive function health perceptions than in individuals without a history of TBI.

Self-reported levels of physical activity have been associated with numerous protective health benefits such as reduced risk of cognitive impairment (Laurin et al., 2001) and cognitive decline (Sofi et al., 2011), mortality due to cardiovascular disease (Nocon et al., 2008) and reduced incident rates of dementia (Larson et al., 2006). Self-report physical activity has also been associated with better health-related quality of life (HRQOL; Brown et al., 2003). These findings from the 2001 behavioral risk factor surveillance system survey found that adhering to recommended levels of physical activity was significantly associated with less days of poor perceived mental and physical health. Our results corroborate these findings in so much as higher physical activity levels are related to higher perceptions of global (mental, physical, social and pain) health. Thereupon, physical activity appears not only associated with improved health outcomes but with higher perceptions of health also.

Numerous randomized-control trials have shown positive associations between physical activity and physical exercise and cognitive function (Colcombe and Kramer, 2003; Gomes-Osman et al., 2018). Additionally, a number of these studies have also shown improvements in the structure and function of cortical networks associated with cognitive functioning (Erickson et al., 2011; Voss et al., 2013; Weng et al., 2017). Whilst dose-response studies are limited (Vidoni et al., 2015; Chen et al., 2018), a linear relationship between physical activity and health status has been reported, such that increases in physical activity leads to greater health benefits, especially in previously sedentary individuals (Warburton et al., 2006). Our results, however, failed to show any association between physical activity levels and perceptions of cognitive functioning in middle to older aged adults free from any neurological or neuropsychiatric disease. This may be explained by the difference in the characteristics of the included participants in our analysis whereby those with a history of TBI had significantly lower NeuroQoL scores compared to those without a history of TBI. This could have been further exacerbated by a ceiling effect in the NeuroQoL scale for the healthy adult population. This scale was developed to assess cognitive complaints in those with neurological afflictions and so may fail to capture a large enough variance in cognitive health perceptions amongst healthy adults (Gershon et al., 2012). Additionally, perceptions of cognitive function may not correlate well with objective measures of cognitive functioning, which is the case in a number of clinical populations (Schiehser et al., 2011; Hutchinson et al., 2012). Consequently, whilst it may be the case that those with lower perceptions of cognitive health receive a greater benefit of adhering to a physically active lifestyle, studies with objective measures of cognitive functioning may further help delineate this relationship.

Some concepts of HRQOL in TBI overlap with those of the general population yet research suggests that HRQOL following TBI may be more complex (Carlozzi et al., 2011). We saw that individuals with a history of TBI had significantly lower self-reported global and cognitive brain health compared to neurologically healthy adults. Whilst we cannot be certain that this lower perception of global and cognitive brain health is derived from the injury, previous reports have shown many individuals with a history of TBI live with residual negative effects of the injury (Benedictus et al., 2010). Cognitive dysfunction is prevalent post-injury and deficits can be seen at 6 months (Dikmen et al., 2009) and for as long as 10 years after injury (Draper and Ponsford, 2008). Long-term lifestyle interventions aimed at reducing these deficits are therefore of great importance to those living with residual effects of TBI.

We found that a similar proportion of those adults with a history of TBI reported being active as those without a history of TBI. However, a numerical majority of participants (both in the history of TBI and healthy group) were classified as insufficiently active. Previous research has suggested that those with a disability or a TBI have distinct barriers to engaging in physical activity (Rimmer et al., 2004; Reavenall and Blake, 2010; Pinto et al., 2018), including but not limited to having health concerns and lack of counselling by a physician (Pinto et al., 2018). Therefore, strategies to increase adherence to a physically active lifestyle, such as the WHO’s Global Strategy on Diet and Physical Activity (Bauman and Craig, 2005), are not only critical for both the general population and community-dwelling adults with a history of TBI but may need to be adapted to those living with a history of TBI. Promising results from a feasibility study of aerobic exercise programs in community-dwelling individuals with a history of moderate-to-severe TBI showed that greater adherence to exercise was achieved when free access to local gymnasiums was provided (Devine et al., 2016). Our results suggest that strategies like these that will increase adherence to a physically active lifestyle, will likely lead to improved perceptions of global and cognitive health.

Our study has certain limitations that may limit the interpretation of the results. Self-reported health outcomes, specifically TBI can be problematic and whilst sports concussion research has improved the self-reporting of concussion through better descriptions of concussion definitions (Robbins et al., 2014), this type of description is less well defined for the general public. Though the accuracy of self-reported measures of certain health outcomes (including stroke) have been documented (Okura et al., 2004), self-reported measures are often the best tool available, especially for large cohort studies. Given this constraint, we did not assess the severity of an individual’s TBI nor the time since injury in our cohort of TBI. Whilst this should not affect the exposure/outcome relationship, it means that we cannot be certain whether different injury severities are more or less associated with the results found. This might be of interest to future studies. Additionally, if co-morbid medical conditions were reported in the history of TBI group, they were not excluded from the analysis. Nevertheless, the number of participants in this group who had multiple conditions was small (headache (Lee et al., 2012), chronic pain (Babyak et al., 2000), anxiety (Gomes-Osman et al., 2018), depression (Blumenthal et al., 1999), memory loss (James et al., 2016), heart attack (Warburton et al., 2006), sleep apnea (Haskell et al., 2007), arthritis (James et al., 2016) and so are unlikely to have significantly affected the results.

## Conclusion

Adhering to a physically active lifestyle is associated with higher global and cognitive health perceptions, especially in individuals with a history of TBI. Notwithstanding, a majority of individuals are insufficiently active and therefore it is critical to develop strategies to increase adherence to and participation in a physically active lifestyle in both those with and without a history of TBI.

## Data Availability

The raw data supporting the conclusions of this manuscript will be made available by the authors, without undue reservation, to any qualified researcher. The datasets generated for this study are available on request to the corresponding author.

## Ethics Statement

This study was carried out in accordance with the recommendations of the ethics and education committee of Institut Guttmann with written informed consent from all subjects. All subjects gave written informed consent in accordance with the Declaration of Helsinki. The protocol was approved by the “Institut Guttmann.”

## Author Contributions

AP-L, DB-F and J-MTM performed the initial conception of the project. GC and JS-S collected the data. TM analyzed the data. TM drafted the manuscript and all authors critically revised it for intellectual content.

## Conflict of Interest Statement

AP-L serves on the scientific advisory boards for Nexstim, Neuronix, Starlab Neuroscience, Neuroelectrics, Constant Therapy, Cognito, and Neosync, and is listed as an inventor on several issued and pending patents on the real-time integration of transcranial magnetic stimulation with electroencephalography and magnetic resonance imaging.

The remaining authors declare that the research was conducted in the absence of any commercial or financial relationships that could be construed as a potential conflict of interest.

## Footnotes

^1^www.bbhi.cat
